# Correlation and prognostic value of SIRT1 and Notch1 signaling in breast cancer

**DOI:** 10.1186/s13046-014-0097-2

**Published:** 2014-11-25

**Authors:** Yu-Wen Cao, Wen-Qin Li, Guo-Xing Wan, Yi-Xiao Li, Xiao-Ming Du, Yu-Cong Li, Feng Li

**Affiliations:** Department of Pathology and Key Laboratory of Xinjiang Endemic and Ethnic Diseases (Ministry of Education), Shihezi University School of Medicine, Shihezi, Xinjiang 832000 China

**Keywords:** SIRT1, N1IC, Snail, Breast cancer, Prognosis

## Abstract

**Background:**

SIRT1 expression and Notch1 signaling have been implicated in tumorigenesis in many cancers, but their association with survival in breast cancer has not been determined. The purpose of this study was to assess the possible prognostic value of SIRT1, N1IC, and Snail expression in breast cancer patients.

**Methods:**

Immunohistochemistry was performed to examine the expression of SIRT1, N1IC, and Snail, and the combined expression of SIRT1 and N1IC, using tissue microarrays containing breast cancer tissue and matched adjacent normal breast tissue from 150 breast cancer patients. Survival analysis was carried out using the Kaplan-Meier method. Univariate and multivariate analysis were used to evaluate the prognostic value of SIRT1, N1IC, Snail and combined SIRT1/N1IC expression, in addition to other clinicopathological factors, including grade, lymph node status, disease stage, and estrogen, progesterone, and human epidermal growth factor receptor 2 receptor status in breast carcinoma patients.

**Results:**

SIRT1, N1IC, and Snail were all found to be highly expressed and an inverse correlation between SIRT1 and N1IC in breast cancer tissue. The three markers significantly correlated with lymph node status. Patients with low SIRT1 expression exhibited shorter overall survival (OS) and disease-free survival (DFS), and patients with combined low expression of SIRT1 and high expression of N1IC had the worse OS and DFS. Univariate and multivariate survival analysis revealed that low expression of SIRT1 and SIRT1-low/N1IC-high expression were independent prognostic factors for poor survival.

**Conclusions:**

These results suggest that low expression of SIRT1 or the combined low expression of SIRT1 and high expression of N1IC could be used as indicators of poor prognosis, and may represent novel therapeutic targets in breast cancer.

**Electronic supplementary material:**

The online version of this article (doi:10.1186/s13046-014-0097-2) contains supplementary material, which is available to authorized users.

## Introduction

Breast cancer is one of the leading causes of cancer mortality in women worldwide. Certain clinicopathological factors such as histological grade, lymph node metastasis, tumor-node-metastasis (TNM) stage, and hormonal status have been widely used to predict clinical outcome. However, due to the heterogeneous nature of the disease, there is no universally applicable prognostic marker for breast cancer. Therefore, the search for novel molecular prognostic biomarkers to identify patients with a very poor prognosis is an ongoing task.

SIRT1 is a type III histone deacetylase. It has also been shown to deacetylate non-histone proteins, including signaling molecules such as Smad [1], STAT3 [2], and c-Myc [3]; the transcription factor P53 [4-6]; and FOXO family proteins [7], which are involved in tumorigenesis, tumor aggression, and prognosis. The role of SIRT1 in breast carcinoma, and especially its association with outcome [5,8-12], is a cause for much debate due to conflicting reports of its dual role as an oncogene [13] and a tumor suppressor gene [14]. Some studies have found that SIRT1 expression is significantly associated with poor survival [8-10], while, in contrast, others reported an association with good prognosis in breast carcinoma [5,11,12]. Therefore, the definitive role of SIRT1 in breast cancer prognosis remains unclear.

Notch1 signaling is a highly conserved communication pathway between neighboring cells. The interaction between the Notch1 receptor and the ligand, Jagged1, Jagged2, or DLL1-3, expressed by adjacent cells, leads to proteolytic cleavage of Notch1 by γ-secretase, thus releasing the Notch1 intracellular domain (N1IC). N1IC then enters the nucleus and regulates downstream gene transcription by binding to transcription factors, such as Snail [15]. Notch1 signaling has been associated not only with varieties of tumors’ proliferation, invasion [16] and prognosis [17] but also with human breast tumorigenesis and progression [18-20], and recent studies have reported that high expression of Notch1 is significantly associated with lymph node metastasis and poor overall survival [21,22]. However, as expression of the Notch1 protein does not always correlate with Notch1 signaling, N1IC is a more reliable marker of activated Notch1 signaling. Our study showed there was not significant difference of Notch1 and N1IC expression in protein or mRNA levels in breast cancer specimen (Additional file 1: Figures S1-S2). We further investigate the N1IC expression of Notch1 signaling and the downstream transcription factor, Snail, with regard to prognosis in breast cancer.

The relationship between SIRT1 expression and Notch1 signaling has been noted in recent years. In non-tumor specimens, the association of SIRT1 with Notch was discussed about stem cell self-renewal, asymmetric cell division, stem cell aging [23], differentiation of neural precursor cells [24], bicuspid aortic valve pathogenesis [25], and vascular growth and energy homeostasis [26,27]. In tumor specimens, previous a study have revealed the Notch signaling was inactivated due to SIRT1 overexpression in Ewing sarcoma cells and offered a novel treatment option in metastatic Ewing sarcoma [28]. In addition, SIRT1 negatively regulated the activity of Notch1 signaling in endothelium of lung cancer and inhibited N1IC expression which leading to endothelial cell proliferation and promoting the growth of lung cancer [14]. However, despite increasing interest in SIRT1 and Notch1 signaling, their expression patterns and prognostic significance in breast carcinoma are unknown.

Therefore, we assessed the possibility of SIRT1 and components of the Notch1 signaling pathway as prognostic biomarkers for breast cancer. This could help to identify patients with poor prognosis who would benefit from additional treatment. In this study, we examined the expression pattern of SIRT1, N1IC, and Snail protein using immunohistochemical staining in breast cancer samples, and investigated their association with clinicopathological parameters. Furthermore, we evaluated the prognostic value of SIRT1 and Notch1 signaling in patients with breast cancer.

## Materials and methods

### Patients and samples

The current study was conducted on 150 patients with breast carcinoma and matched adjacent normal breast tissues. These cases were obtained from the First Affiliated Hospital, Shihezi University, School of Medicine and were diagnosed between January 2000 and December 2009. Immunohistochemical (IHC) analysis of formalin-fixed paraffin-embedded specimens was carried out at the hospital’s Department of Pathology. The staining results were independently evaluated by two experienced pathologists, without prior knowledge of clinical information. Multiple clinical and pathological parameters were obtained from medical records and original pathology reports, including age (range 29–80 years), histological grade (1 and 2 versus 3), lymph node metastasis (absent versus present), TNM stage (I, II, and III versus IV), estrogen receptor (ER), progesterone receptor (PR), and human epidermal growth factor receptor 2 (HER2) status (negative versus positive), and use of adjuvant treatment (chemotherapy, radiotherapy and endocrine therapy). In the evaluation of IHC results of HER2, negative (−) and (1+) were regarded as negative status, and (3+) was regarded as positive status, to (2+) IHC result, it was further assessed the positive or negative status using FISH. The follow up for 150 patients was carried out during clinic interviews or with phone calls, because of survival data missing on 28 patients, therefore, a final number of 122 cases breast cancer patients’ survival state were analyzed in the present study. The total period of follow-up was 2–161 months. The matched normal breast tissues were collected at least 4 cm away from the tumor site. The study was approved by the Institutional Human Ethics Committee of Shihezi University School of Medicine, and written consent was received from all patients enrolled in the study.

### Immunohistochemistry on tissue microarrays

We reviewed all hematoxylin and eosin stained slides and selected the appropriate breast cancer area for preparation of the tissue microarray sections. Then one core (1.0 mm in diameter) of representative areas from each cancer in paraffin block were deposited in a new paraffin block using a semi-automated tissue arrayer. The sections were cut from tissue microarray paraffin blocks, dewaxed in xylene, and rehydrated in graded alcohol. Antigen retrieval was carried out in EDTA (pH 9.0; Zymed, Life Technologies, Carlsbad, CA, USA) using microwave. Endogenous peroxidase activity was blocked by immersion in 3% hydrogen peroxide at room temperature for 10 min. Tissue sections were incubated at 4°C overnight with anti-SIRT1 (1:200, sc-15404; Santa Cruz Biotechnology, Dallas, TX, USA), anti-N1IC (1:200, ab8925; Abcam, Cambridge, UK), or anti-Snail (1:50; ab53519; Abcam, Cambridge, UK). Sections were subsequently incubated with secondary antibody (Dako Cytomation EnVision System, Dako, Denmark) for 30 min and visualization was performed using DAB (Dako, Denmark). Finally, tissue sections were counterstained with hematoxylin. Sections were washed with PBS twice for 5 min between each step. The primary antibody was replaced with PBS for control experiments.

### Evaluation of immunostaining

Expression levels of SIRT1, N1IC, and Snail were semi-quantitatively scored by calculating the percentage of positively stained cells and the staining intensity, according to Wu et al. [9] and Jethwa P et al. [29], with slight modifications. The percentage of positively stained cells was scored on a scale of 0 to 4 as follows: 0 (<1%, absent), 1 (1–24%, sporadic), 2 (25–49%, local), 3 (50–74%, majority), and 4 (75–100%, vast majority). The staining intensity was scored from 0 to 3 as follows: 0 (negative), 1 (buff), 2 (yellow), and 3 (brown). The scores for percentages of positive cells and staining intensities were then multiplied to generate an immunoreactivity score (IS) for each case. The IS ranged from 0–12 (0, 1, 2, 3, 4, 6, 8, 9, and 12). Cutoff values for this scoring system were assigned as follows: high expression of SIRT1, N1IC, and Snail was defined as an IS of ≥4 (4, 6, 8, 9, and 12); and low expression was defined as an IS of <4 (0, 1, 2, and 3).

### Statistical analysis

The correlation between N1IC, SIRT1, and Snail expression was determined using Spearman’s rank correlation analysis. Inter-relationships between the three markers and clinicopathological parameters were assessed using contingency tables, with a two-tailed chi-squared test or Fisher’s exact test for trend analysis, as appropriate. Survival curves were estimated using the Kaplan–Meier method and compared using the log rank test. Overall survival (OS) time was calculated from the date of surgery to the date of death or to the end of follow-up. Disease-free survival (DFS) time was calculated from the date of surgery to the documented date of disease progression (relapse or metastasis) or to the end of follow-up. Univariate and multivariate survival analyses with calculation of hazard ratios (HR) were performed using Cox’s proportional-hazards model to assess whether a factor was an independent predictor of OS or DFS. All statistical analyses were performed using the SPSS software system (version 17.0; SPSS, Inc., Chicago, IL, USA) and GraphPad Prism 5.01 (GraphPad Software Inc., La Jolla, CA, USA). A *p*-value ≤0.05 was considered to be statistically significant.

## Results

### Expression of SIRT1, N1IC, and snail protein in breast carcinoma tissue

**Figure 1:**
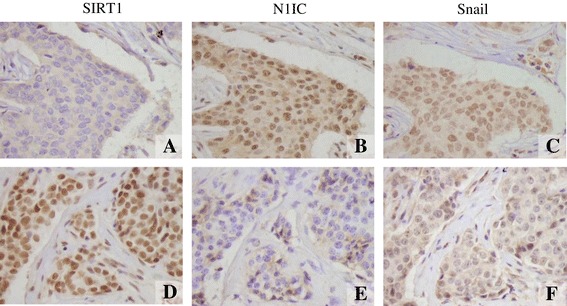
**Representative images of immunohistochemical staining of SIRT1, N1IC and snail in breast carcinoma tissues.** All markers are primarily expressed in the nuclei of the tumor cells. (original magnification × 400). **A)**, **B)**, and **C)**: Low expression of SIRT1 and high expression of N1IC, Snail in consecutive slides and co-localization. **D)**, **E)**, and **F)**: High expression of SIRT1 and low expression of N1IC, Snail in consecutive slides and co-localization. **A)** and **D)**: Low and high expression of SIRT1. **B)** and **E)**: High and low expression of N1IC. **C)** and **F)**: High and low expression of Snail.

We measured the protein expression of SIRT1, N1IC, and Snail in 150 patients with breast cancer and matched adjacent normal breast tissues. The positive staining (low vs. high) photographs of SIRT1, N1IC and Snail protein in cancer tissues were presented in Figure 1 using the consecutive slides and co-localization. SIRT1 and Snail were predominantly localized in the nucleus, while N1IC staining was located in the nucleus and/or cytoplasm. Only nuclear N1IC expression was evaluated.

**Figure 2:**
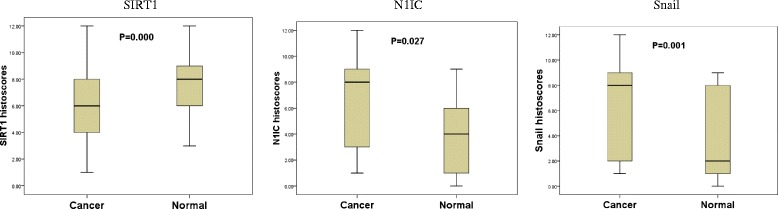
**The protein expression levels of SIRT1, N1IC, and Snail in 150 cases with breast cancer and adjacent normal tissues.**

The expression profiles of SIRT1, N1IC, and Snail in cancer tissues compared with corresponding normal tissues were presented in Figure 2. The results showed the expression level of SIRT1 protein was significantly lower in cancer than normal tissues (*p* = 0.000), then, the expression level of N1IC and Snail were markedly up-regulated in cancer tissues (*p* = 0.027, *p* = 0.001, respectively).

In all cancer and normal tissues, the expression of SIRT1, N1IC, and Snail were presented mainly in high expression status (Table 1). Moreover, the high expression rate of SIRT1 was significantly lower in cancer than normal tissues (*p* = 0.000). On the contrary, the high expression rate of N1IC and Snail were markedly increasing in cancer tissue (*p* = 0.003, *p* = 0.001, respectively).

**Table 1:** **The expression status of SIRT1, N1IC and sail in 150 patients with breast cancer and adjacent normal breast tissues**

**Group**	**n**	**SIRT1**		***p-*** **value**	**N1IC**		***p-*** **value**	**Snail**		***p-*** **value**
		**High (%)**	**Low (%)**		**High (%)**	**Low (%)**		**High (%)**	**Low (%)**	
Cancer	150	116 (77.3)	34 (22.7)	0.000*	112 (75.0)	38 (25.0)	0.003*	93 (62.0)	57 (38.0)	0.001*
Normal	150	138 (92.0)	12 (8.0)		88 (58.7)	62 (41.3)		65 (43.3)	85 (56.7)	

*P < 0.05.

**Figure 3:**
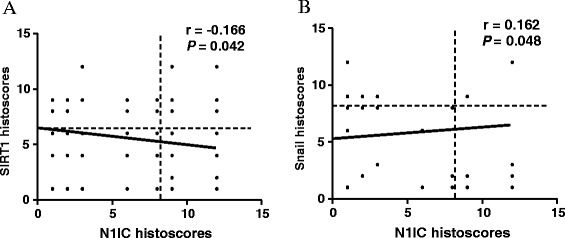
**The pairwise comparison between SIRT1, N1IC, and Snail expression in cancer tissues. A)** The association of SIRT1 with N1IC expression. **B)** The association of N1IC with Snail expression.

The expression relationship between SIRT1 and N1IC protein in 150 samples with breast cancer showed a significant inverse correlation (r = −0.166, *p* = 0.042) in statistical analysis (Figure 3A). In contrast, we found that N1IC expression had a markedly positive association (r = 0.162, *p* = 0.048) with Snail expression in cancer tissues (Figure 3B).

### Relationship between expression of SIRT1, N1IC, snail and clinicopathological characteristics

We performed the correlation analysis between the expression of SIRT1, N1IC, Snail, and clinicopathological variables in 150 breast cancer patients (Table 2). The results showed that low expression of SIRT1 was markedly associated with lymph node metastasis (*p* = 0.023) and negative PR status (*p* = 0.021). However, the high expression of N1IC and Snail was significantly related to lymph node metastasis (*p* = 0.001, *p* = 0.029, respectively).

**Table 2:** **Association of expression level of SIRT1, N1IC, and snail with clinicopathological variables**

		**SIRT1**				**N1IC**				**Snail**			
**Characteristics**	**N**	**High**	**Low N (%)**	***χ*** ^***2***^	***p-*** **value**	**High N (%)**	**Low N (%)**	***χ*** ^***2***^	***p*** **-value**	**High N (%)**	**Low N (%)**	***χ*** ^***2***^	***p*** **-value**
Age (years)													
< 50	74	57 (77.0)	17 (23)	0.008	0.930	60 (81.1)	14 (18.9)	3.177	0.075	49 (66.2)	25 (33.8)	1.102	0.294
≥ 50	76	59 (77.6)	17 (22.4)			52 (68.4)	24 (31.6)			44 (57.9)	32 (42.1)		
Lymph node metastasis													
Absence	83	70 (84.3)	13 (15.7)	5.2	0.023*	53 (63.9)	30 (36.1)	11.48	0.001*	45 (54.2)	38 (45.8)	4.778	0.029*
Presence	67	46 (68.7)	21 (31.3)			59 (88.0)	8 (12.0)			48 (71.6)	19 (28.4)		
TNM stage													
I–III	128	98 (76.6)	30 (23.4)	0.3	0.788	94 (73.4)	34 (26.6)	1.457	0.227	76 (59.4)	52 (40.6)	2.552	0.110
IV	22	18 (81.8)	4 (18.2)			18 (81.8)	4 (18.2)			17 (77.3)	5 (22.7)		
Histological grade													
1 + 2	102	81 (79.4)	21 (20.6)	0.79	0.375	73 (71.6)	29 (28.4)	1.617	0.203	60 (58.8)	42 (41.2)	1.365	0.243
3	48	35 (72.9)	13 (27.1)			39 (81.3)	9 (18.7)			33 (68.8)	15 (31.2)		
ER status													
Negative	71	53 (74.6)	18 (25.4)	0.56	0.456	51 (71.8)	20 (28.2)	0.573	0.449	44 (62.0)	27 (38.0)	0	0.995
Positive	79	63 (79.7)	16 (20.3)			61 (77.2)	18 (22.8)			49 (62.0)	30 (38.0)		
PR status													
Negative	71	49 (69.0)	22 (31.0)	5.32	0.021*	52 (73.2)	19 (26.8)	0.145	0.703	41 (57.7)	30 (42.3)	1.035	0.309
Positive	79	67 (84.8)	12 (15.2)			60 (75.9)	19 (24.1)			52 (65.8)	27 (34.2)		
HER2 status													
Negative	111	23 (20.7)	88 (79.3)	0.92	0.337	32 (28.8)	79 (71.2)	2.758	0.097	44 (39.6)	67 (60.4)	0.487	0.485
Positive	39	11 (28.2)	28 (71.8)			6 (15.4)	33 (84.6)			13 (33.3)	26 (66.7)		
Adjuvant therapy													
Chemo	75	57 (76.0)	18 (24.0)	0.268	0.966	57 (76.0)	18 (24.0)	1.313	0.726	47 (63.1)	28 (36.9)	5.853	0.119
Chemo + Radio	15	12 (80.0)	3 (20.0)			13 (86.7)	2 (13.3)			13 (86.7)	2 (13.3)		
Chemo + Radio + Endo	12	9 (75.0)	3 (25.0)			9 (75.0)	3 (25.0)			9 (75.0)	3 (25.0)		
Unknown	48	38 (79.2)	10 (20.8)			35 (72.9)	13 (27.1)			27 (56.3)	21 (43.7)		

Chemo: chemotherapy; Radio: radiotherapy; Endo: endocrine therapy; N: number of patients; *P < 0.05.

In addition, we classified breast cancer patients into four groups according to the combined expression status of SIRT1 and N1IC as follows: SIRT1-high/N1IC-low (n = 34); SIRT1-low/N1IC-low (n = 5); SIRT1-high/N1IC-high (n = 81); and SIRT1-low/N1IC-high (n = 30). The relationship between these groups and clinicopathological variables was analyzed (Table 3). The SIRT1-low/N1IC-high group was associated with negative HER2 status (*p* = 0.018) and combination therapy of chemotherapy, radiotherapy and endocrine (*p* = 0.042).

**Table 3:** **Association of combined expression status of SIRT1 and N1IC with clinicopathological parameters**

**Characteristics**	**N**	**SIRT1/N1IC**					
		**High/low**	**Low/low**	**High/high**	**Low/high**	***χ*** ^***2***^	***p-*** **value**
		**N (%)**	**N (%)**	**N (%)**	**N (%)**		
Age (years)							
<50	74	12 (16.2)	1 (1.4)	44 (59.4)	17 (23.0)	6.027	0.110
≥50	76	22 (28.9)	4 (5.3)	37 (48.7)	13 (17.1)		
Lymph node metastasis							
Absence	83	18 (21.7)	4 (4.8)	48 (57.8)	13 (15.7)	3.664	0.300
Presence	67	16 (23.9)	1 (1.5)	33 (49.3)	17 (25.4)		
TNM stage							
I-III	128	30 (23.4)	4 (3.1)	69 (53.9)	25 (19.5)	0.441	0.932
IV	22	4 (18.2)	1 (4.5)	12 (54.5)	5 (22.7)		
Histological grade							
1 + 2	102	18 (17.6)	4 (3.9)	55 (53.9)	25 (24.5)	7.334	0.062
3	48	16 (33.3)	1 (2.1)	26 (54.2)	5 (10.4)		
ER status							
Negative	71	17 (23.9)	2 (2.8)	33 (46.5)	19 (26.8)	4.728	0.193
Positive	79	17 (21.5)	3 (3.8)	48 (60.8)	11 (13.9)		
PR status							
Negative	71	17 (23.9)	2 (2.8)	36 (50.7)	16 (22.5)	0.910	0.823
Positive	79	17 (21.5)	3 (3.8)	45 (57.0)	14 (17.7)		
HER2 status							
Negative	111	28 (25.2)	4 (3.6)	52 (46.8)	27 (24.3)	10.052	0.018*
Positive	39	6 (15.4)	1 (2.6)	29 (74.4)	3 (7.7)		
Adjuvant therapy							
Chemo	75	17 (22.7)	2 (2.7)	43 (57.3)	13 (17.3)	17.431	0.042*
Chemo + radio	15	2 (13.3)	1 (6.7)	7 (46.7)	5 (33.3)		
Chemo + radio + endocrine	12	2 (16.7)	1 (8.3)	2 (16.7)	7 (58.3)		
Unknown	48	13 (27.1)	1 (2.1)	29 (60.4)	5 (10.4)		

Chemo: chemotherapy; Radio: radiotherapy; Endo: endocrine therapy; N: number of patients; *P < 0.05.

### Association of SIRT1, N1IC, and snail expression with prognosis in breast cancer

**Figure 4:**
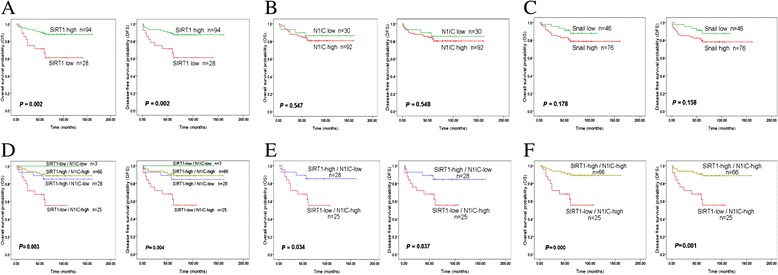
**Survival analyses for SIRT1, N1IC and Snail expression in breast cancer patients. A)** Association of expression level of SIRT1 with OS and DFS. **B)** Association of expression level of N1IC with OS and DFS. **C)** Association of expression level of Snail with OS and DFS. **D)** Association of combined expression status of SIRT1 and N1IC with OS and DFS. **E)** Association of SIRT1-low/N1IC-high and SIRT1-high/N1IC-low with OS and DFS. **F)** Association of SIRT1-low/N1IC-high and SIRT1-high/N1IC-high with OS and DFS.

The prognostic impact of SIRT1 expression and Notch1 signaling was also analyzed in 122 of all 150 patients with breast carcinoma (because of the missing survival data in 28 patients). The relationship of SIRT1, N1IC, and Snail expression with OS and DFS was investigated using Kaplan–Meier survival curves (Figure 4). We found that low expression of SIRT1 significantly correlated with poor prognosis (*p* = 0.002 for both OS and DFS) (Figure 4A). Expression of N1IC and Snail did not display statistical difference with OS and DFS (Figure 4B,C).

In addition, we analyzed the prognostic significance of the combined expression of SIRT1 and N1IC. Patients with SIRT1-low/N1IC-high expression had significantly shorter OS (*p* = 0.003) and DFS (*p* = 0.004) compared to the patients in other three group (Figure 4D). The SIRT1-low/N1IC-high group was also association with markedly decreasing of OS (*p* = 0.034) and DFS (*p* = 0.037) when comparing to the SIRT1-high/N1IC-low group (Figure 4E). Additionally, the SIRT1-low/N1IC-high was significantly related to poorer survival (OS: *p* = 0.000; DFS: *p* = 0.001) comparing with SIRT1-high/N1IC-high group (Figure 4F). However, no significant difference in OS and DFS was found between SIRT1-low/N1IC-high and SIRT1-low/N1IC-low group, it’s may be too few patients (n = 3) in the SIRT1-low/N1IC-low group. There also was no significant difference when paired in comparison in other groups.

Univariate Cox regression analysis was performed to analyze the relationship between SIRT1, N1IC, Snail expression, clinicopathological features and OS and DFS in 122 breast carcinoma patients (Table 4). Consistent with the Kaplan-Meier survival curves, patients with low expression of SIRT1 alone and the SIRT1-low/N1IC-high expression had significantly shorter OS and DFS. Moreover, the SIRT1-low/N1IC-high group had shorter OS (HR, 3.278-fold) and DFS (HR, 3.233-fold) than the low expression of SIRT1 alone (OS: HR, 0.278-fold; DFS: HR, 0.282-fold). The expression level of N1IC or Snail was not significantly associated with OS and DFS. Of the clinicopathological features analyzed, lymph node metastasis, TNM stage IV, and negative ER and PR status were significantly related to shorter OS and DFS.

**Table 4:** **Univariate Cox regression analysis for overall survival (OS) and disease-free survival (DFS) in 122 breast cancer patients**

**Variables**	**OS**			**DFS**		
	**HR**	**95% CI**	***p-*** **value**	**HR**	**95% CI**	***p-*** **value**
Breast cancer patients (n =122)						
Age (≥50 vs. <50)	1.631	0.676–3.935	0.277	1.611	0.688–3.888	0.288
Lymph node metastasis (presence vs. absence)	9.111	2.681–30.961	0.000*	9.123	2.685–30.997	0.000*
TNM stage (IV vs. 0–III)	6.851	2.901–16.180	0.000*	7.255	3.067–17.162	0.000*
Histological grade (3 vs. 1 + 2)	1.145	0.462–2.837	0.771	1.149	0.464–2.848	0.764
ER status (negative vs. positive)	0.338	0.131–0.871	0.025*	0.351	0.136–0.901	0.031*
PR status (negative vs. positive)	0.186	0.063–0.553	0.002*	0.191	0.064–0.566	0.003*
HER2 status (positive vs. negative)	0.657	0.241–1.793	0.412	0.646	0.237–1.763	0.393
SIRT1 expression (low vs. high)	0.278	0.118–0.657	0.004*	0.282	0.121–0.666	0.004*
N1IC expression (high vs. low)	1.395	0.469–4.145	0.549	1.394	0.469–4.142	0.551
Snail expression (high vs. low)	1.966	0.719–5.372	0.188	2.031	0.743–5.552	0.167
SIRT1/N1IC expression (high/low)		1	0.013		1	0.014
SIRT1/N1IC expression (low/low vs. high/low )	0	0	0.985	0	0	0.985
SIRT1/N1IC expression (high/high vs. high/low)	0.693	0.203-2.368	0.559	0.692	0.203-2.366	0.557
SIRT1/N1IC expression (low/high vs. high/low)	3.278	1.025–10.481	0.045*	3.233	1.012–10.326	0.048*

*P < 0.05.

To address whether the expression level of SIRT1and N1IC or combined expression status of SIRT1/N1IC was an independent prognostic factor in 122 patients with breast cancer, we performed multivariate survival analysis using two models (Table 5). Model 1 analyzed the expression profiles of SIRT1, N1IC, Snail, and clinical variables. Model 2 was adjusted for combined expression patterns of SIRT1/N1IC instead of expression of SIRT1 and N1IC alone. The results revealed that low expression of SIRT1, lymph node metastasis, and negative PR status were independent prognostic factors of shorter OS and DFS (Table 5, Model 1). The expression of SIRT1-low/N1IC-high, lymph node metastasis, and negative PR status were also independent prognostic indicators of shorter OS and DFS (Table 5, Model 2). Moreover, the SIRT1-low/N1IC-high group has shorter OS and DFS (HR, 3.519-fold and 3.613-fold, respectively) than those with low expression of SIRT1 alone (OS: HR, 0.220-fold; DFS: HR, 0.207-fold).

**Table 5:** **Multivariate Cox regression analysis for overall survival (OS) and disease-free survival (DFS) in 122 breast cancer patients**

**Variables**	**OS**			**DFS**		
	**HR**	**95% CI**	***p*** **-value**	**HR**	**95% CI**	***p*** **-value**
**Model 1 Breast cancer patients (n =122)**						
LN metastasis (presence vs. absence)	9.078	2.269–36.322 2.269–36.322	0.002*	9.061	2.262–36.296 2.262–36.296	0.002*
TNM stage (IV vs. 0–III)	1.652	0.612–4.460	0.322	1.769	0.645–4.847	0.268
ER status (negative vs. positive)	1.193	0.347–4.101	0.779	1.130	0.332–3.845	0.845
PR status (negative vs. positive)	0.165	0.039–0.688	0.013*	0.179	0.043–0.752	0.019*
SIRT1 expression (low vs. high)	0.220	0.079–0.614	0.004*	0.207	0.074–0.58	0.003*
N1IC expression (high vs. low)	0.815	0.249–2.675	0.736	0.793	0.24–2.621	0.704
Snail expression (high vs. low)	1.189	0.639–5.174	0.262	1.938	0.677–5.551	0.218
**Model 2 Breast cancer patients (n =122)**						
LN metastasis (presence vs. absence)	7.913	2.002–31.286	0.003*	7.777	1.965–30.786	0.003*
TNM stage (IV vs. 0–III)	1.659	0.614–4.483	0.318	1.798	0.654–4.943	0.256
ER status (negative vs. positive)	1.203	0.353–4.093	0.768	1.152	0.342–3.882	0.819
PR status (negative vs. positive)	0.166	0.040–0.685	0.013*	0.180	0.043–0.749	0.018*
Snail expression (high vs. low)	1.901	0.667–5.418	0.229	2.031	0.709–5.821	0.187
SIRT1/N1IC expression (high/low)		1	0.023		1	0.016
SIRT1/N1IC expression (low/low vs. high/low)	0	0	0.986	0	0	0.987
SIRT1/N1IC expression (high/high vs. high/low)	0.695	0.202-2.395	0.564	0.665	0.191-2.317	0.522
SIRT1/N1IC expression (low/high vs. high/low)	3.519	1.031–12.01	0.045*	3.613	1.066–12.25	0.039*

*P < 0.05.

## Discussion

Recent studies of the prognostic role of SIRT1 in breast cancer have reported contradictory results [8-12], and previous studies concerning the role of Notch1 signaling in breast carcinoma have reported that expression of Notch1 is related to poor survival [21,22], but to date, no direct assessment of the correlation between SIRT1 expression, Notch1 signaling, and patient outcome has been carried out. To this end we examined the immunohistochemical expression of SIRT1, N1IC, and Snail and analyzed their prognostic significance in breast carcinoma. Our results suggest that, of these proteins, only the low expression of SIRT1 alone, and the combined expression of SIRT1-low/N1IC-high are significantly associated with worse survival in breast cancer patients.

The prognostic role of SIRT1 in human tumors has previously been studied. Jung et al. [30] reported that SIRT1 over-expression is a favorable prognostic factor for colorectal cancer. Recent studies in breast carcinoma have found SIRT1 to be an indicator of good prognosis [12]. It has also been reported to be involved in suppressing cancer cell growth, invasion, and metastasis, by deacetylation of Bcl-2 [31], p53 [5] and c-Myc [11], and affecting estrogen signaling [32]. In our study, SIRT1 was highly expressed in 77.3% (116/150) breast cancer patients, and SIRT1 over-expression was associated with prolonged survival. Regression analysis using the Cox’s proportional hazards model confirmed that low expression of SIRT1 was associated with a poorer prognosis. Moreover, multivariate analysis indicated the low expression of SIRT1 was a statistically significant indicator of poor prognosis, independent of established clinicopathological prognostic factors. In addition, the low expression of SIRT1 was associated with lymph node metastasis and negative PR status. Therefore we believed that low expression of SIRT1 identifies a group of tumors with a very poor prognosis, and suggests that SIRT1 may be a potential therapeutic target in breast cancer. However, in contrast to our results and other previous reports, some studies have indicated that over-expression of SIRT1 may correlate with poor prognosis in certain types of tumor [33-36], including breast carcinoma [8-10]. This discrepancy might arise from differences in tissue specificity or differences in downstream targets of the enzyme. Therefore, further research is needed to clarify the function of SIRT1.

Notch1 signaling has been proposed as a poor prognostic marker in breast carcinoma [21,22]. Notch1 is involved in migration and invasion of tumor cells, and an elevated Notch1 protein is associated with poor outcome [19]. However, the detection of the Notch1 protein alone cannot determine whether Notch1 signaling is active. Therefore, we performed additional immunostaining for N1IC, the intracellular form of Notch1 protein, and marker of activated Notch1 signaling. As expected, our results showed the high level of N1IC expression (75.0%, 112/150) in breast tumor specimens was consistent with our previous findings of up-regulated Notch1 in breast cancer tissue [37]. Our study demonstrated that N1IC over-expression was associated with poor prognosis using Kaplan-Meier analyses. However, there was no statistical correlation of N1IC with survival by univariate Cox regression analysis, and N1IC was not found to be an independent prognostic factor by multivariate analysis. With regard to the relationship between N1IC and clinicopathological parameters, we found that high expression of N1IC was significantly related to lymph node metastasis, which is in agreement with our previous studies focusing on Notch1, meanwhile, over-expression of Notch1 was positively correlated with invasion and metastasis by epithelial-mesenchymal transition (data not shown). In addition, the transcription factor Snail was highly expressed in breast cancer patients (62%, 93/150), but the high expression of Snail was not significantly related to poor survival of patients, then, it was associated with lymph node metastasis. This was consistent with published findings that up-regulation of Snail promoted mammary tumor cell migration, invasion, and metastasis via RANKL inducing epithelial-mesenchymal transition [38]. Meanwhile, the expression of Snail was a positive correlation with N1IC expression, which is consistent with previous studies carried out in hepatocellular carcinoma [15,39]. This result underlined the important synergistic effect of N1IC and Snail proteins on breast cancer progression, and also confirmed the finding that demonstrated in other scholar studies [40,41]. In next study, we will need to explore possible target genes of N1IC or Snail, which may be suitable for therapeutics of breast cancer. In current, just only the role of Notch1 or N1IC protein in cancer therapies have been reported [42,43], which demonstrated that rescue of notch1 signaling in antigen-specific CD8+ T cells enhances immunotherapy in cancer [42], and γ-secretase (in Notch signaling) inhibitor PF-03084014 and docetaxel (activating Notch pathway) can synergistically effect on therapy of breast cancer [43].

The most striking research in our study was the evaluation of correlation between SIRT1 protein and Notch1 signaling with breast cancer prognosis. Several recent studies have suggested that SIRT1 could be a negative regulator of endothelial Notch signaling through N1IC low expression in angiogenesis [26,14]. Guarani et al. [26] reported that SIRT1 acted as a negative modulator of Notch1 signaling in the level of N1IC protein in endothelial cells. The study showed that deacetylation of NICD (Notch1 intracellular domain) on conserved lysines by deacetylase SIRT1 resulted in down-regulating of acetylated NICD, and the decreasing acetylated N1IC underwent more ubiquitin-mediated degradation because acetylation can impair ubiquitination, and caused low N1IC protein level. Subsequently, the expression of target genes in Notch1 signaling were decreased, as a consequence, which increased vascular branching and density. Furthermore, Mian Xie et al. [14] also indicated that SIRT1 negatively regulated Notch1 signaling mainly in the level of N1IC protein stability in endothelial cell of lung cancer. Their results demonstrated that deacetylation of promoter region in Notch1 by SIRT1 lead to repressing of Notch1 transcription and reducing of acetylated N1IC. Subsequently, N1IC protein levels was decreasing, and repressed the expression of Notch1 target genes, eventually enhanced tumor neovascularization and promoted lung tumor growth. Our findings were in accordance with the observations that the negative association of SIRT1 with N1IC in breast cancer tissues. Our results showed SIRT1 and N1IC protein expression was significantly inverse correlation in cancer tissues from 150 patients. In addition, we analyzed the association of SIRT1 with N1IC expression only in the 122 cases used for OS and DFS (Additional file 2: Figure S3). The results were in accordance with the observations in 150 cases. Moreover, we further analyzed the combined expression status of the two markers with disease outcome. The results showed that SIRT1-low/N1IC-high expression was associated with shorter OS and DFS compared with SIRT1-high/N1IC-low group, which further suggested an inverse correlation between SIRT1 and N1IC in breast cancer prognosis.

More important, we need further to evaluate the impacting prognosis of SIRT1 and N1IC expression level (high vs. low) and the two markers expression pattern (combined vs. alone) in breast cancer tissues. Our results showed the that combined SIRT1-low/N1IC-high expression was an independent worst prognostic factor in all SIRT1/N1IC groups and it associated with negative HER2 status and combined adjuvan therapy of chemotherapy, radiotherapy and endocrine therapy, which indicated SIRT1-low/N1IC-high may serve as a key poor prognostic and therapeutic indicator. Meanwhile, as mentioned above, we found low expression of SIRT1 alone was an independent factor for poor prognosis. In addition, we also found the SIRT1-low/N1IC-high group has shorter OS and DFS than those with low expression of SIRT1 alone. Taken together, these findings suggested high SIRT1 may be breast cancer protective in a Notch1-depndent manner. Further, we analyzed these results and found it was the interesting that 25 out of 28 SIRT1 low expression cancers were N1IC high expression, combining with shorter OS and DFS in SIRT1-low/N1IC-high group than in low SIRT1 alone, so the finding raised the intriguing possibility that inhibition of N1IC in low SIRT1 breast tumors might improve the survival of patients. Additionally, when compared the prognosis relationship between SIRT1-low/N1IC-high and SIRT1-high/N1IC-high group, our results showed SIRT1-low/N1IC-high group had worse outcome than the SIRT1-high/N1IC-high group, which confirmed low expression of SIRT1 may be an important indicator for worse survival in N1IC high breast cancer. However, no significant difference of OS or DFS were found in other groups, which may be partly due to the too few registered patients in SIRT1-low/N1IC-low subgroup (3 cases). All the results together, the SIRT1 protein and Notch1 signaling mainly manifested in the level of N1IC played an important role in the prognosis of patients, and may represent therapeutic targets for breast cancer. The mechanism by which SIRT1 represses N1IC expression will be needed to further be explored.

## Conclusion

In conclusion, this study has demonstrated that low expression of SIRT1 was significantly associated with poor outcome, and combined low expression of SIRT1 and high expression of N1IC could identify breast cancer patients with the worst prognosis. Protein expression of SIRT1 and N1IC showed a significant inverse correlation. These findings suggest that the SIRT1-Notch1 signaling axis is important in breast cancer progression, and SIRT1 may be a more important prognostic biomarker in Notch1-depndent manner to breast carcinoma. The mechanism of SIRT1 and Notch1 signaling in breast cancer progression needs to be further studied in order to harness the potential of SIRT1and N1IC expression in the clinical setting.

## Additional files

Additional file 1:
**Protein and mRNA expression profiles of Notch1 and N1IC in breast tissues.**
Additional file 2:
**The association of SIRT1 with N1IC expression in 122 breast cancer samples.**

## Abbreviations

DFS: Disease free survival
ER: Estrogen receptor
FISH: Fluorescence *in situ* hybridization
HER2: Human epidermal growth factor receptor 2
HR: Hazard ratio
IHC: Immunohistochemical
IS: Immunoreactivity score
N1IC: Notch1 intracellular domain
OS: Overall survival
PBS: Phosphate-buffered saline
PR: Progesterone receptor
TNM: Tumor-node-metastasis
