# Supplementary material for: Correlation and prognostic value of SIRT1 and Notch1 signaling in breast cancer
Source: J Exp Clin Cancer Res. 2014 Nov 25;33(1):97. doi: 10.1186/s13046-014-0097-2 (PMC4248440; doi:10.1186/s13046-014-0097-2)
Supplement: Additional file 1: — Protein and mRNA expression profiles of Notch1 and N1IC in breast tissues. [file 13046_2014_97_MOESM1_ESM.doc]

**Additional file 1**

We performed the expression comparison on protein and mRNA levels between Notch1 and N1IC in breast cancer specimen, respectively.

(1) The high expression of Notch1 protein in breast carcinoma was 85.2% (69/81), which had been reported in our previous study [1]. To compare the protein expression of Notch1 and N1IC, in present study we choose out 81 of all 150 cases of N1IC expression, which corresponding to the same patient of Notch1 expression. The results showed that the high expression of N1IC protein was 75.3% (61/81). These data revealed there was no significant difference (*p*=0.114) of protein expression between Notch1 and N1IC. The finding indicated parallel expression level of Notch1 and N1IC protein.

(2) To compare the mRNA expression of Notch1 and N1IC, we conducted additional experiments for mRNA expression of Notch1 and N1IC using quantitative real-time polymerase chain reaction (qPT-PCR) in 81 cases with breast cancer corresponding to same patients of its protein expression. The adjacent normal breast tissues were used as control. These primers were used: Notch1: forward: 5′-GGA CCT CAT CAA CTC ACA CG-3′, reverse: 5′-GGT GTC TCC TCC CTG TTG TT-3′ [2]; N1IC: forward: 5′-ATG TTC TTT GTG GGC TGT GGG-3′, reverse: 5′- TGG CAG TGA TGT TGG TAG GGC-3′ [3]. The experiment condition was as previously described [4]. The thermal cycling program was applied: 10 min at 95℃, 40 cycles of 15 s at 95℃ and 1min at 60℃. Data normalization was accomplished using the endogenous controlβ-actin and the normalized values were subjected to a 2^-ΔΔCt^ formula to calculate the fold change between the breast cancer and control normal groups. We found the markedly higher expression of Notch1 mRNA (*p* = 0.000) and N1IC mRNA (*p* = 0.000) in cancer than in normal tissue (Figure S1). Furthermore, we analyzed the association of Notch1with N1IC mRNA expression in cancer tissues. The results demonstrated there was no significant difference (*p* = 0.066) between Notch1 and N1IC mRNA levels (Figure S2). We speculated the expression level of Notch1 mRNA may be not regulated by other factors in breast cancer tissues.

**A**

**B**

**Figure S1.** Expression level of Notch1 and N1IC mRNA in breast cancer and normal breast tissue.

A) The mean mRNA level of Notch1 in cancer tissues was 2.79-fold significantly higher than in normal tissues (p = 0.000).

B) The mean mRNA level of N1IC in cancer tissues was 2.55-fold significantly higher than in normal tissues (p = 0.000).


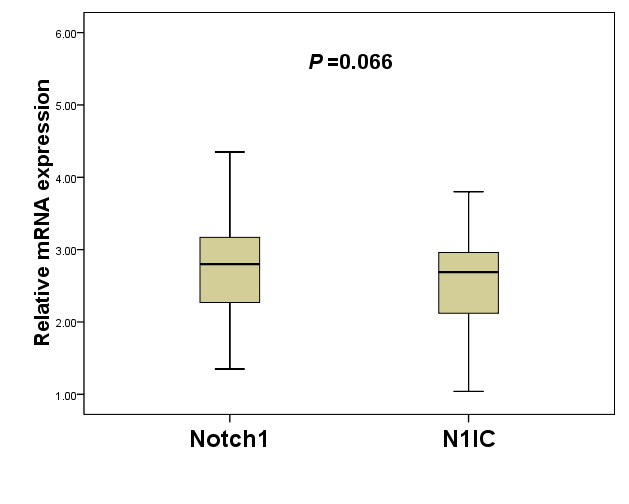
 **Figure S2.**  The comparison of mRNA expression between Notch1 and N1IC gene in breast cancer tissues. The results showed it was no significant difference (p=0.066) that mRNA expression of Notch1 compared with N1IC in breast cancer tissues.

References

1．Yu-Wen Cao, Guo-Xing Wan, Chun-Xia Zhao, et al: **Notch1 single nucleotide polymorphism rs3124591 is associated with the risk of development of invasive ductal breast carcinoma in a Chinese population.** *Int J Clin Exp Pathol* 2014, 7(7):4286-4294

2．Dara Nalls, Su-Ni Tang, Marianna Rodova, et al: **Targeting Epigenetic Regulation of miR-34a for Treatment of Pancreatic Cancer by Inhibition of Pancreatic Cancer Stem Cells.** *PloS One* 2011, 6(8):e24099.

3．Mian Xie, Ming Liu, Chao-Sheng He, et al: **SIRT1 Regulates Endothelial Notch Signaling in Lung cancer.** *PloS One* 2012, 7(9):e45331.

4．Liu C, Li D, Jiang J, Hu J, et al: **Analysis of molecular cytogenetic alteration in rhabdomyosarcoma by array comparative genomic hybridization.** *PLoS One* 2014, 9(4):e94924.
