# Supplementary material for: Correlation and prognostic value of SIRT1 and Notch1 signaling in breast cancer
Source: J Exp Clin Cancer Res. 2014 Nov 25;33(1):97. doi: 10.1186/s13046-014-0097-2 (PMC4248440; doi:10.1186/s13046-014-0097-2)
Supplement: Additional file 2: — The association of SIRT1 with N1IC expression in 122 breast cancer samples. [file 13046_2014_97_MOESM2_ESM.doc]

**Additional file 2**

**Figure S3.** The association of SIRT1 with N1IC expression in 122 breast cancer samples. The results showed a significant inverse correlation between SIRT1 and N1IC protein (r = -0.275, *p* = 0.002).
